# Pregnancy outcomes and prognostic factors after history- and ultrasound-indicated cerclage in women with cervical insufficiency and/or short cervix: A retrospective cohort study

**DOI:** 10.1016/j.eurox.2026.100489

**Published:** 2026-09-07

**Authors:** Cecile C. Hulshoff, Maria G. Plasman, Kanthi de Groot, Jacklijn A.A. Pijls, Freke A. Wilmink, Marc E.A. Spaanderman, Ralph R. Scholten, Joris van Drongelen

**Affiliations:** a Department of Obstetrics and Gynecology, Radboud University Medical Center, Nijmegen, the Netherlands; b Department of Obstetrics and Gynecology, Maastricht University Medical Center, Maastricht, the Netherlands

**Keywords:** cerclage, cervical insufficiency, cervical length, elective, echo-indicated, history-indicated, premature birth, prophylactic, ultrasound-indicated

## Abstract

**Objective:**

This study evaluated pregnancy outcomes after history- and ultrasound-indicated cerclage and predictors of gestational age at delivery and pregnancy prolongation.

**Method:**

11-year retrospective cohort study in a tertiary center in the Netherlands. Women were included with singleton pregnancies receiving history- or ultrasound-indicated cerclage for suspected cervical insufficiency and/or short cervix < 24 + 0 weeks. Analyses were stratified by cerclage indication. Ultrasound-indicated group was subdivided by obstetric history (with suspected prior cervical insufficiency [UIC-A] or without [UIC-B]). Kaplan–Meier analysis and univariable linear regression were used to assess associations between pre-/post-cerclage factors and gestational outcomes.

**Results:**

A total of 272 women received history-indicated and 79 ultrasound-indicated cerclage (UIC-A: 36, UIC-B: 43). Median gestational age at delivery was comparable (37.9–38.4 weeks). Among women with suspected prior cervical insufficiency, spontaneous preterm birth < 34 weeks was significantly less frequent after history-indicated than ultrasound-indicated cerclage (12% vs 25%), with comparable perinatal survival. Among ultrasound-indicated cases, UIC-A showed higher rates of spontaneous preterm birth < 34 weeks (25% vs 7%) and preterm premature rupture of membranes (31% vs 9%). Greater post-cerclage distance between external os and cerclage was associated with higher gestational age at delivery and pregnancy prolongation, whereas pre-cerclage measures showed little association.

**Conclusions:**

Pregnancy outcomes after cerclage differ according to clinical context and cerclage indication. In women with suspected prior cervical insufficiency, ultrasound-indicated cerclage after cervical shortening identified a subgroup at higher risk of spontaneous preterm birth and pregnancy complications. Post-cerclage cervical parameters were the strongest predictors. Optimizing cerclage placement height may be associated with improved outcomes.

## 1 Introduction

Preterm birth (PTB) affects one in ten live births worldwide and remains the leading cause of neonatal mortality [1,2]. Various mechanisms contribute to spontaneous PTB (sPTB). Extreme PTB (<28 weeks of gestation) accounts for 4.2% of all PTB, but has the highest neonatal mortality and morbidity [1,3,4]. Cervical insufficiency (CI) is an important, potentially treatable contributor to extreme sPTB. CI is characterized by recurrent painless cervical dilation, leading to second-trimester loss or extreme PTB, and accounts for 15–20% of perinatal losses between 16–28 weeks [[5], [6], [7], [8]].

Treatment strategies for CI include non-surgical approaches and surgical cervical cerclage, in which a suture or tape is placed around the cervix to provide mechanical support. Cerclage prolongs pregnancy and reduces recurrent PTB [9,10]. Depending on clinical indication, placement may follow cervical dilation (emergency cerclage), cervical shortening (ultrasound-indicated cerclage), or an obstetric history of sPTB (history-indicated cerclage) [8,[11], [12], [13]].

In women with an obstetric history suggestive of CI, both history- (HIC) and ultrasound-indicated cerclage (UIC) are used, but optimal selection, timing, and prognostic stratification remain uncertain. Previous studies suggest similar rates of sPTB and neonatal outcomes, but are limited by small sample sizes, heterogeneous CI definitions, varying sPTB cut-offs, and underpowered subgroup analyses [[14], [15], [16], [17], [18], [19]]. Safety outcomes are inconsistently reported, and few studies have explored prognostic factors that might explain outcome differences within and between these groups. This limits the ability to define optimal selection criteria, particularly among women with complex or mixed risk factors.

The aim of this study was to evaluate pregnancy outcomes following cervical cerclage in routine clinical practice, considering indication as clinical context rather than aiming to determine superiority between strategies. We compared outcomes between women receiving HIC or UIC in the context of an obstetric history suggestive of CI and/or a cervical length < 25 mm before 24^+0^ weeks of gestation. Outcomes were compared after UIC with and without a prior obstetric history suggestive of CI. We also evaluated pre- and post-cerclage factors associated with gestational age (GA) at delivery and pregnancy prolongation to support individualized counselling and clinical decision-making.

## 2 Methods

### 2.1 Study design

We conducted a retrospective observational cohort study of women with singleton pregnancies undergoing HIC or UIC at a tertiary care center (Radboud University Medical Center, Nijmegen, the Netherlands) between November 2013 and July 2024.

HIC was placed at approximately 12–14 weeks of gestation in women with an obstetric history suggestive of CI, defined as ≥ 1 sPTB between 16–28 weeks of gestation and/or previous successful transvaginal cerclage. UIC was placed < 24^+0^ weeks of gestation in women, with or without history suggestive of CI, with a shortened cervix; ≤ 25 mm was used as threshold. In women without a history suggestive of CI, routine cervical length screening was not performed; a short cervix was detected incidentally during routine obstetric care or evaluation of symptoms, either at our centre or prior to referral. Cerclage was not offered in case of threatened preterm labour, chorioamnionitis, PPROM, fetal demise, or fetal anomalies. Women with abdominal or emergency cerclage, multifetal pregnancies, or iatrogenic PTB after cerclage placement were excluded. GA, fetal anomalies, and cervical length measurements pre- and post-cerclage were assessed by ultrasonography.

### 2.2 Surgical technique

**Fig. 1:**
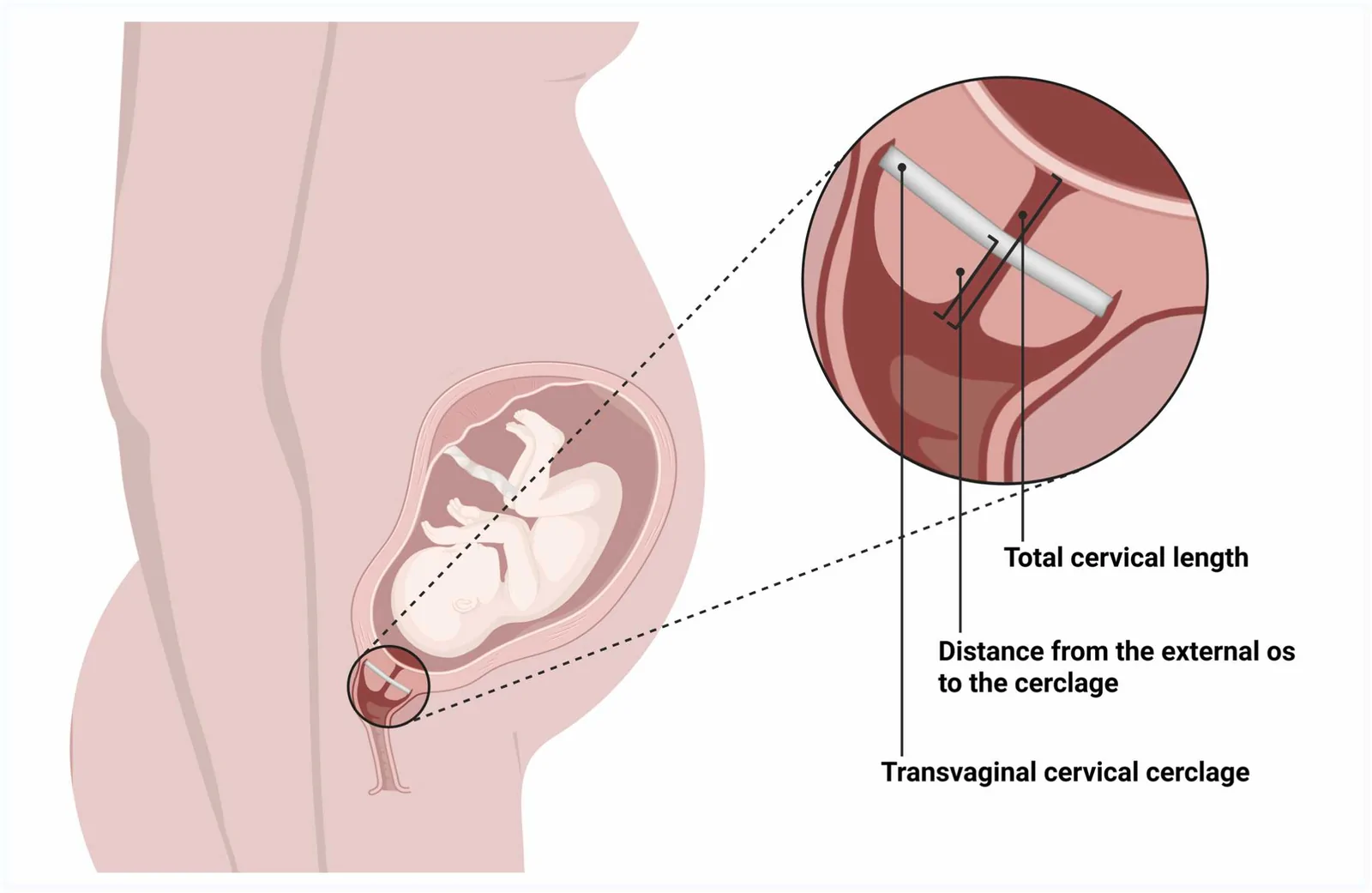
Schematic representation of a transvaginal cervical cerclage and post-cerclage cervical measurements, including the distance from the external os to the cerclage and the total cervical length.

Cerclage placement was performed under spinal or general anaesthesia according to standard surgical/perioperative protocols by a perinatologist or clinical fellow. The cervico-vesical mucosa was opened and the bladder was retracted, in line with the Shirodkar technique, to allow cerclage placement approximately 20–25 mm from the external os. The cerclage was placed using the four-step technique with a 5-mm Mersilene tape (Fig. 1) [11]. All women received prophylactic antibiotics (cefazolin 1000 mg). Perioperative tocolysis with nifedipine and/or indomethacin was administered according to local protocol and physician discretion. Standard out-patient follow-up visits were scheduled 2–3 weeks after cerclage placement. Follow-up included transvaginal ultrasound assessment of cervical length and cerclage position. Cerclages were removed around 36 weeks of gestation, or earlier if indicated by threatened sPTB, cervical tearing, or clinical signs of chorioamnionitis.

### 2.3 Outcomes

Women undergoing HIC or UIC were identified through operation records. Data were retrospectively collected by the primary investigator via chart review. Baseline characteristics included maternal age, surgical and obstetric history, and cervical characteristics at diagnosis. Primary outcome was GA at delivery. Secondary maternal outcomes included perioperative complications, admissions for threatened sPTB, pregnancy prolongation, PPROM occurring at any time after cerclage placement, mode of delivery, and postpartum complications. Neonatal outcomes included Apgar scores, birthweight, and perinatal survival. In accordance with Dutch national practice, active neonatal management is generally initiated from 24^+0^ weeks of gestation. Perinatal survival outcomes were therefore analysed separately for pregnancies delivered before and after 24 + 0 weeks of gestation.

### 2.4 Statistical analysis

Analyses were performed within predefined groups [1]: HIC, and [2] UIC. The latter was further stratified into women with (A) a history suggestive for CI, i.e. previous cerclage and/or sPTB between 16–28 weeks of gestation (UIC-A), and (B) without such history (UIC-B). Comparisons were conducted between [1] HIC versus UIC-A, and [2] UIC-A versus UIC-B. Group comparisons were performed using Student *t* test, χ² test, Fisher exact test, or Mann–Whitney *U* test, as appropriate. For between-group comparisons, effect estimates with 95% confidence intervals were additionally calculated. Odds ratios with 95% confidence intervals were reported for categorical outcomes, while differences in medians with bootstrap 95% confidence intervals were reported for continuous outcomes. A p-value < 0.05 was considered significant.

Kaplan–Meier survival analyses were used to assess prolongation of pregnancy after placement. Survival curves were stratified by relevant clinical variables, including cerclage indication and cervical length cut-offs, and compared using the log-rank test. No censoring occurred, as all pregnancies ultimately resulted in delivery during follow-up. Exploratory univariable linear regression analyses were performed to assess pre- and post-cerclage factors associated with GA at delivery and pregnancy prolongation.

Exploratory subgroup analyses were performed to assess whether stratification by cervical length influenced pregnancy outcomes. Another exploratory subgroup analysis was conducted to assess whether changes in cervical length and the time-interval of these changes prior to UIC influenced GA at delivery. Linear regression models included absolute cervical length change, weekly change, and their interaction with the time interval. A secondary model additionally included baseline cervical length at 10–12 weeks of gestation. Only women with cervical length measurements available at both 10–12 weeks and immediately pre-cerclage were analysed. Given the limited sample size, these analyses were considered exploratory. No adjustment for multiple testing was applied; subgroup and regression analyses were interpreted as exploratory. Analyses were performed using available cases, without imputation of missing data.

Analyses were performed using R software version 4.4.1 [[20], [21], [22], [23], [24]].

## 3 Results

From November 2013 until July 2024 we placed 356 HIC and UIC in singleton pregnancies. Five women were excluded as they had an iatrogenic PTB < 37^+0^ weeks (indications included pre-eclampsia, renal failure, placenta praevia), resulting in 351 women included for analysis.

### 3.1 Baseline characteristics of women treated with HIC or UIC

Table 1 presents the baseline characteristics. A total of 272 women underwent HIC and 79 underwent UIC during the study period. In the UIC-group, 36 had a history suspected for CI (UIC-A) and 43 women did not (UIC-B). Prior cervical procedures were performed less often in women in the HIC (6%) compared to the UIC-group (14%). Significantly more women in the HIC-group (44%) had received a previous vaginal cerclage compared to the UIC-A group (6%).

**Table 1:** Baseline characteristics of women treated with history- or ultrasound-indicated cerclage.

	1. History-indicated cerclage (HIC)	2. Ultrasound-indicated cerclage (UIC)			p-value	
		A. With history suspected for CI	B. Without history suspected for CI	Total (UIC)	HIC – UIC-A	UIC-A – UIC-B
*Demographic data*						
Number of participants	272	36	43	79		
Age (years)	32.0 (6.0)	32.0 (7.0)	32.0 (6.5)	32.0 (6.0)	0.33	0.69
Uterus anomaly (%)	15 (6%)	1 (3%)	5 (12%)	6 (8%)	0.70	0.21
1st^-^grade family member with (suspected) cervical insufficiency (%)	28 (10%)	3 (8%)	6 (14%)	9 (11%)	1.00	0.49
BMI (kg/m^2^)	25.6 (7.6)	25.8 (4.2)	24.2 (6.4)	25.0 (5.2)	0.83	0.52
*Surgical history*						
Uterine surgery (%)^a^	105 (39%)	14 (39%)	15 (35%)	29 (37%)	1.00	0.82
Cervical surgery (%)^b^	17 (6%)	4 (11%)	7 (16%)	11 (14%)	0.29	0.53
*Obstetric history*						
Gravidity (number per subject)	3.0 (2.0)	3.0 (1.0)	3.0 (2.0)	3.0 (2.0)	**0.01**	0.98
Parity (number per subject)^c^	2.0 (1.0)	1.0 (0.0)	1.0 (2.0)	1.0 (0.0)	**< 0.01**	0.67
Nulliparous (%)	0 (0%)	0 (0%)	14 (33%)	15 (19%)	1.00	**< 0.01**
Previous offspring survival (%)	189 / 317 (60%)	21 / 47 (47%)	53 / 61 (87%)	74 / 108 (69%)	0.06	**< 0.01**
Previous transvaginal cerclage (%)	119 (44%)	2 (6%)	0 (0%)	2 (3%)	**< 0.01**	**0.01**

CI, cervical insufficiency; GA, gestational age.

Data are presented as median (IQR) or n (%). Bold indicates statistical significance, with significance set at p-value < 0.05.

^a^, caesarean section, myoma enucleation, septum resection, curettage, hysteroscopy.

^b^, LLETZ or conization.

^c,^ Delivery ≥ 16 weeks, independent of outcome, counting twin deliveries as one.

### 3.2 Perioperative characteristics, interventions and complications during pregnancy after HIC or UIC

Table 2 presents perioperative characteristics and complications within two weeks after cerclage placement. Median GA at placement was 13.9 weeks for HIC. For UIC, median GA at placement was 21.4 weeks, comparable for UIC-A and UIC-B. Median cervical length pre-cerclage was 39.0 mm in the HIC-group. In the UIC-group, median cervical length pre-cerclage was 16.5 mm, comparable between UIC-A and UIC-B. Perioperative PPROM occurred in one woman (3%) in the UIC-A group, within 24 h after cerclage placement. Cervical laceration perioperative occurred in two women in the HIC (1%) and UIC-B group (2%). No other perioperative complications, including bladder lesions, were observed.

**Table 2:** Perioperative characteristics and complications after placement of the history- or ultrasound-indicated cerclage.

	1. History-indicated cerclage (HIC)	2. Ultrasound-indicated cerclage (UIC)			p-value	
		A. With history suspected for CI	B. Without history suspected for CI	Total (UIC)	HIC – UIC-A	UIC-A – UIC-B
*Perioperative characteristics and care*						
GA at cerclage placement (weeks)	13.9 (1.1)	20.5 (4.5)	21.7 (2.2)	21.4 (1.5)	**< 0.01**	0.07
Cervical length pre-cerclage (mm)^a^	39.0 (7.0)	19.0 (11.0)	15.0 (9.0)	16.5 (10.0)	**< 0.01**	0.23
*Perioperative complications*						
Failure to place cerclage (%)	0 (0%)	0 (0%)	0 (0%)	0 (0%)	1.00	1.00
Perioperative PPROM (%)	0 (0%)	1 (3%)	0 (0%)	0 (0%)	0.11	0.46
Other perioperative complications (%)^b^	1 (1%)	0 (0%)	1 (2%)	1 (1%)	1.00	1.00

CI, cervical insufficiency; GA, gestational age; PPROM, preterm premature rupture of membranes.

Data are presented as median (IQR) or n (%). Bold indicates statistical significance, with significance set at p-value < 0.05.

^a^, Data not available for all patients in the history-indicated group: N, 276.

^b^, other perioperative complications include blood loss > 500 ml, bladder lesion, cervical laceration or tear.

Table 3 presents interventions and complications during pregnancy. A re-cerclage was placed < 24^+0^ weeks of gestation in eight women after HIC (3%): in five women due to tearing of the cerclage, in three women due to suboptimal placement based on ultrasound examination. After UIC-A, a re-cerclage was placed in two women (3%), due to suboptimal placement. Median total cervical length 2–4 weeks after (re)cerclage placement was 42.0 mm after HIC, median distance between external os and cerclage was 24.0 mm. After UIC, median total cervical length was 33.0 mm, comparable between subgroups UIC-A and UIC-B. Distance between external os and cerclage was 21.0 mm in both the total UIC-group and subgroups. PPROM occurred in 39 women (14%) after HIC. After UIC, PPROM occurred in 15 women (19%), significantly more often after UIC-A compared to UIC-B (31% vs 9%, respectively). Infection was suspected based on clinical and elevated infectious parameters in 23 women (8%) after HIC, and in 15 (19%) after UIC. After initial admission for cerclage placement, 99 women (36%) were readmitted for threatening sPTB after HIC and 46 women (58%) after UIC. Cervical lacerations due to cerclage tearing were observed in 25 women (9%) after HIC, at a median of 30.4 weeks of gestation. After UIC, lacerations were observed in 9 women (11%): in 14% of women after UIC-A and 9% after UIC-B. These lacerations occurred at a non-significant earlier GA after UIC-A compared to UIC-B (27.0 vs 34.9 weeks, respectively), and were identified during examination following uterine contractions or routine check-ups without contractions. Lacerations were small, rarely requiring minor additive surgery.

**Table 3:** Interventions and complications during pregnancy after placement of the history- or ultrasound-indicated cerclage.

	1. History-indicated cerclage (HIC)	2. Ultrasound-indicated cerclage (UIC)			p-value	
		A. With history suspected for CI	B. Without history suspected for CI	Total (UIC)	HIC – UIC-A	UIC-A – UIC-B
*Interventions*						
Transvaginal re-cerclage (%)	8 (3%)	2 (6%)	0 (0%)	2 (3%)	0.33	0.20
− Due to tearing of cerclage (%)	5 (62%)	0 (0%)	NA	0 (0%)	0.44	NA
− Due to suboptimal placement (%)	3 (38%)	2 (100%)	NA	2 (100%)	0.44	NA
Cervical length 2–4 weeks post-(re)cerclage (mm)						
− Total cervical length (mm)	42.0 (9.0)	35.0 (9.0)	31.0 (8.0)	33.0 (8.5)	**< 0.01**	0.18
− Distance between external os and cerclage (mm)	24.0 (5.0)	21.0 (6.0)	21.0 (6.5)	21.0 (5.5)	**< 0.01**	0.84
Therapeutic antibiotics (%)	19 (7%)	8 (22%)	6 (14%)	14 (18%)	**0.01**	0.39
Therapeutic tocolysis (%)^a^	5 (2%)	1 (3%)	0 (0%)	1 (1%)	0.52	0.46
*Complications*						
PPROM (%)	39 (14%)	11 (31%)	4 (9%)	15 (19%)	**0.03**	**0.02**
Infection (suspected) (%)	23 (8%)	8 (22%)	7 (16%)	15 (19%)	**0.02**	0.57
Hospital readmissions for threatening PTB (%)	99 (36%)	23 (64%)	23 (53%)	46 (58%)	**< 0.01**	0.37
− Number of readmissions − Hospital stay at readmission (days)	0.0 (1.0) 4.0 (4.0)	1.0 (0.3) 4.0 (6.0)	1.0 (1.0) 4.0 (5.5)	1.0 (1.0) 4.0 (6.0)	**< 0.01** 0.27	0.18 0.49
Cervical laceration due to tearing of cerclage (%)	25 (9%)	5 (14%)	4 (9%)	9 (11%)	0.37	0.72
− GA at cervical laceration due to tearing of cerclage (weeks)	30.4 (9.6)	27.0 (12.1)	34.9 (2.5)	28.7 (7.6)	0.56	0.07
− Prolongation after tearing of cerclage (days)	2.0 (22.0)	2.0 (9.0)	20.0 (23.3)	13.0 (13.0)	0.71	0.06

CI, cervical insufficiency; PPROM, preterm premature rupture of membranes; PTB, preterm birth; GA, gestational age.

Data are presented as median (IQR) or n (%). Bold indicates statistical significance, with significance set at p-value < 0.05.

^a^, other than prophylactic maintenance dose of Adalat oros (individualized dose, up until 60 mg/12 h) and/ or indomethacin (individualized dose, up until 50 mg/ 4 h).

### 3.3 Pregnancy outcomes after HIC or UIC

Table 4 presents pregnancy outcomes after cerclage placement. Median GA at delivery was 38.4 weeks after HIC and 37.9 weeks after UIC. sPTB < 34 weeks of gestation occurred in 12% after HIC and in 15% after UIC. sPTB < 34 weeks of gestation occurred most often after UIC-A: significantly more frequent after UIC-A compared to HIC (25% vs 12%), and compared to UIC-B (25% vs 7%). sPTB < 32, < 28, and < 24 weeks were 11%, 7%, and 5% after HIC, and 11%, 5%, and 3% after UIC, respectively, comparable between UIC subgroups. Median prolongation of pregnancy between cerclage placement and delivery was 172.0 days after HIC and 116.0 days after UIC. Overall survival was 94% after HIC, with fetal and neonatal survival of 99% and 96%, respectively. For pregnancies that passed beyond 24^+0^ weeks, viable fetal and neonatal survival were 100% and 99%, respectively. After UIC, overall survival was 97%, with fetal and neonatal survival of 100% and 97%, respectively, similar across UIC-A and UIC-B. For pregnancies that passed beyond 24^+0^ weeks, viable survival was 100%. Birthweight of viable neonates was lowest after UIC-A (3040 g), significantly lower than HIC (3250 g) and non-significantly lower than UIC-B (3120 g).

**Table 4:** Pregnancy outcomes after placement of the history- or ultrasound-indicated cerclage.

	1. History-indicated cerclage (HIC)	2. Ultrasound-indicated cerclage (UIC)						
					**Effect estimate**	**p-value**	**Effect estimate**	**p-value**
		A. With history suspected for CI	B. Without history suspected for CI	Total (UIC)	HIC – UIC-A	UIC-A – UIC-B		
*Obstetric outcomes*								
GA at delivery (weeks)	38.4 (2.6)	37.9 (4.7)	38.0 (2.4)	37.9 (3.2)	0.6 (−0.3–2.3)	0.19	−0.1 (−2.1–1.1)	0.40
Delivery ≥ 34 weeks of GA (%)	240 (88%)	27 (75%)	40 (93%)	67 (85%)	2.49 (0.94–6.09)	**0.04**	0.23 (0.04–1.03)	**0.03**
sPTB < 34 weeks of GA (%)	32 (12%)	9 (25%)	3 (7%)	12 (15%)	0.40 (0.16–1.06)	**0.04**	4.36 (0.97–27.32)	**0.03**
sPTB < 32 weeks of GA (%)	29 (11%)	6 (17%)	3 (7%)	9 (11%)	0.60 (0.22–1.91)	0.27	2.63 (0.51–17.60)	0.29
sPTB < 28 weeks of GA (%)	20 (7%)	2 (6%)	2 (5%)	4 (5%)	1.35 (0.30–12.41)	1.00	1.20 (0.08–17.41)	1.00
sPTB < 24 weeks of GA (%)	13 (5%)	1 (3%)	1 (2%)	2 (3%)	1.75 (0.25–76.74)	1.00	1.20 (0.01–96.34)	1.00
Pregnancy prolongation (days)	172.0 (24.0)	116.0 (35.3)	115.0 (25.0)	116.0 (31.5)	56.0 (49.0–68.0)	< 0.001	1.0 (−14.5–10.0)	0.78
Mode of delivery − Vaginal delivery (%) − Caesarean section (%)	215 (79%) 57 (21%)	26 (72%) 10 (28%)	29 (67%) 14 (33%)	55 (70%) 24 (30%)	1.45 (0.59–3.33) 0.69 (0.30–1.70)	0.39 0.39	1.25 (0.43–3.74) 0.80 (0.27–2.33)	0.81 0.81
Postpartum maternal complications − Infection (%) − Sepsis (%) − Postpartum hemorrhage > 1 L (%)	21 (8%) 1 (1%) 23 (8%)	5 (14%) 1 (3%) 4 (11%)	4 (9%) 0 (0%) 2 (5%)	9 (11%) 1 (1%) 6 (8%)	0.52 (0.17–1.89) 0.13 (0.00–10.43) 0.74 (0.23–3.13)	0.21 0.22 0.54	1.56 (0.31–8.58) NE 2.53 (0.34–29.66)	0.72 0.46 0.40
*Neonatal outcomes*								
Survival								
− Overall:	257 / 272 (94%)	35 / 36 (97%)	42 / 43 (98%)	77 / 79 (97%)	0.49 (0.01–3.38)	0.70	0.84 (0.01–67.23)	1.00
• Fetal survival rate (%)^a^	268 / 272 (99%)	36 / 36 (100%)	43 / 43 (100%)	79 / 79 (100%)	NE	1.00	NE	1.00
• Neonatal survival (%)^b^	257 / 268 (96%)	35 / 36 (97%)	42 / 43 (98%)	77 / 79 (97%)	0.67 (0.02–4.86)	1.00	0.84 (0.01–67.23)	1.00
− Fetuses born < 24 weeks of GA:	0 / 13 (0%)	0 / 1 (0%)	0 / 0 (0%)	0 / 1 (0%)	NE	1.00	NA	NA
• Non-viable fetal survival < 24 weeks (%)^a^	9 / 13 (69%)	1 / 1 (100%)	NA	1 / 1 (100%)	NE	1.00	NA	NA
• Non-viable neonatal survival < 24 weeks (%)^b^	0 / 9 (0%)	0 / 1 (0%)	NA	0 / 1 (0%)	NE	1.00	NA	NA
− Fetuses born ≥ 24 weeks of GA:	257 / 259 (99%)	35 / 35 (100%)	42 / 43 (98%)	77 / 78 (99%)	NE	1.00	NE	1.00
• Viable fetal survival rate ≥ 24 weeks (%)^a^	259 / 259 (100%)	35 / 35 (100%)	43 / 43 (100%	78 / 78 (100%)	NE	1.00	NE	1.00
• Viable neonatal survival ≥ 24 weeks (%)^b^	257 / 259 (99%)	35 / 35 (100%)	42 / 43 (98%)	77 / 78 (99%)	NE	1.00	NE	1.00
Apgar scores								
− At 1 min	9.0 (1.0)	9.0 (1.0)	9.0 (2.0)	9.0 (1.0)	0.0 (0.0–0.5)	0.78	0.0 (−0.5–0.0)	0.89
− At 5 min	10.0 (1.0)	10.0 (1.0)	10.0 (1.0)	10.0 (1.0)	0.0 (0.0–1.0)	0.63	0.0 (−1.0–0.0)	0.54
Birthweight (g)^c^	3250 (780)	3040 (937)	3120 (897)	3050 (940)	210 (−21–622)	0.01	−80 (−643–223)	0.18

CI, cervical insufficiency; GA, gestational age; sPTB, spontaneous preterm birth; OR, odds ratio; NE, not estimable due to zero cells; NA, not applicable.

Data are presented as median (IQR) or n (%).Bold indicates statistical significance, with significance set at p-value < 0.05.

Effect estimates are presented as ORs with 95% CIs for categorical outcomes and differences in medians with bootstrap 95% CIs for continuous outcomes.

a, Based on fetuses alive at birth.

b, Based on neonates alive at the 28th day after birth.

c, Birthweight of viable fetus.

**Fig. 2:**
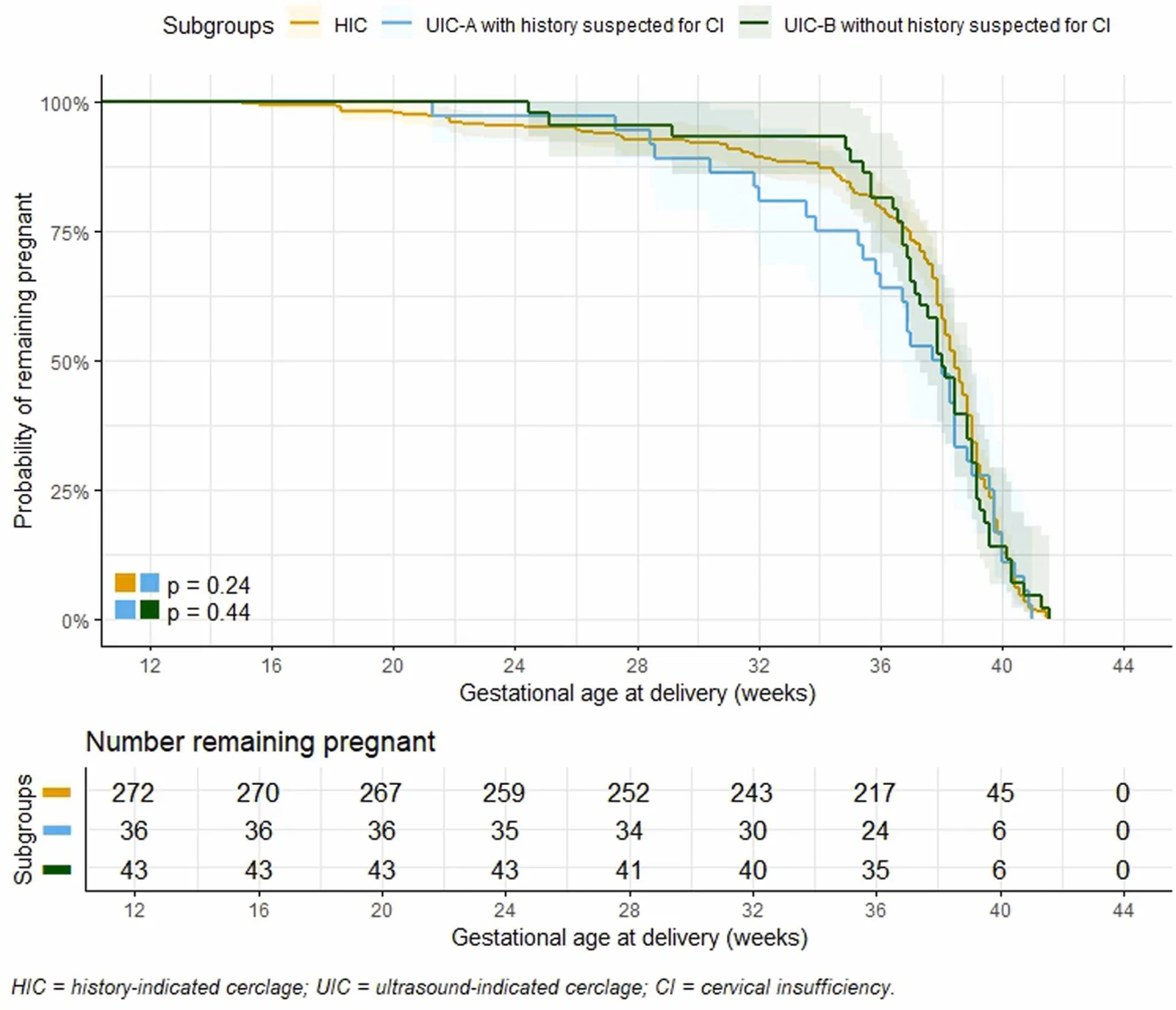
Kaplan Meier curve of the percentage of women remaining pregnant during the course of pregnancy after placement of the history- or ultrasound-indicated cerclage

Fig. 2 shows Kaplan–Meier curves. No significant differences were observed in the proportion of women remaining pregnant after cerclage placement between HIC and UIC-A, or between UIC-A and UIC-B.

Exploratory subgroup analyses stratified by pre-cerclage cervical length and obstetric history showed broadly comparable outcomes between groups (Appendices A–D).

### 3.4 Regression-analyses of factors associated with pregnancy outcomes after HIC or UIC

Table 5, Table 6 present exploratory univariable regression analyses of factors associated with GA at delivery and pregnancy prolongation. Pre-cerclage factors were not consistently associated with either outcome across groups, whereas post-cerclage parameters showed more consistent associations. Post-cerclage cervical length was positively associated with GA at delivery in both UIC subgroups and with pregnancy prolongation in the UIC-A group. Distance between the external os and the cerclage was associated with higher GA at delivery and greater pregnancy prolongation in the HIC-group and UIC subgroups.

**Table 5:** Univariable linear regression analyses of factors associated with gestational age at delivery (days).

	**History-indicated cerclage**			**Ultrasound-indicated cerclage**					
				**UIC-A: With history suspected for CI**			**UIC-B: Without history suspected for CI**		
	**β (95% CI)**	**SE**	**P**	**β (95% CI)**	**SE**	**P**	**β (95% CI)**	**SE**	**P**
**Before cerclage**									
**GA at cerclage placement (weeks)**	0.08 (-0.40; 0.57)	0.24	0.73	4.2 (0.63; 7.84)	1.75	**0.02**	-0.77 (-4.48; 2.94)	1.82	0.67
**BMI pre-gravid (kg/m²)**	0.01 (-0.09; 0.12)	0.05	0.84	-3.36 (-6.23; −0.49)	1.40	**0.02**	0.28 (-1.47; 2.03)	0.84	0.74
**Maternal age (years)**	-0.07 (-0.21; 0.06)	0.07	0.27	-1.26 (-3.29; 0.84)	0.98	0.23	1.12 (-0.56; 2.80)	0.84	0.19
**Cervical length before cerclage (mm)**	0.05 (-0.05; 0.14)	0.05	0.32	-0.07 (-1.61; 1.47)	0.77	0.91	-0.35 (-1.68; 0.98)	0.63	0.60
**After cerclage**									
**Total cervical length after (re)cerclage (mm)**	7.64 (-2.65; 17.94)	5.23	0.15	1.82 (0.42; 3.29)	0.70	**0.02**	1.19 (0.07; 2.31)	0.56	**0.04**
**Distance external os to cerclage (mm)**	19.71 (32.79; 6.64)	6.64	**< 0.01**	4.83 (1.68; 7.98)	1.54	**< 0.01**	2.80 (1.12; 4.55)	0.84	**< 0.01**

CI, confidence interval; GA, gestational age; BMI, Body Mass Index; CI, cervical insufficiency.

Bold indicates statistical significance, with significance set at p-value < 0.05**.**

**Table 6:** Univariable linear regression analyses of factors associated with prolongation (days) after cerclage placement.

	**History-indicated cerclage**			**Ultrasound-indicated cerclage**					
				**UIC-A: With history suspected for CI**			**UIC-B: Without history suspected for CI**		
	**β (95% CI)**	**SE**	**P**	**β (95% CI)**	**SE**	**P**	**β (95% CI)**	**SE**	**P**
**Before cerclage**									
**BMI pre-gravid (kg/m²)**	0.10 (-0.66; 0.85)	0.38	0.80	-1.97 (-4.78; 0.83)	1.37	0.16	0.74 (-1.34; 2.81)	1.03	0.48
**Maternal age (years)**	-0.80 (-1.75; 0.15)	0.48	0.10	-1.51 (-3.45; 0.43)	0.95	0.12	1.02 (-1.07; 3.10)	1.03	0.33
**Cervical length before cerclage (mm)**	0.22 (-0.45; 0.88)	0.34	0.52	0.16 (-1.33; 1.64)	0.73	0.83	-0.36 (-1.96; 1.24)	0.79	0.65
**After cerclage**									
**Total cervical length after (re)cerclage (mm)**	7.36 (-3.35; 18.06)	5.43	0.18	1.50 (0.08; 2.92)	0.70	**0.04**	1.19 (-0.17–2.55)	0.67	0.09
**Distance external os to cerclage (mm)**	20.57 (7.07; 34.07)	6.85	**< 0.01**	3.75 (0.57; 6.94)	1.56	**< 0.01**	3.40 (1.39–5.42)	1.00	**< 0.01**

CI, confidence interval; GA, gestational age; BMI, Body Mass Index; CI, cervical insufficiency.

Bold indicates statistical significance, with significance set at p-value < 0.05.

Exploratory analyses of pre-UIC cervical length changes showed no clear associations with GA at delivery (Appendix E).

## 4 Discussion

### 4.1 Main findings

Prevention of extreme sPTB remains a major clinical priority. In this 11-year observational cohort, outcomes were compared between HIC and UIC in women with a history suspected for CI (UIC-A), and between UIC in women with (UIC-A) versus without a history suspected for CI (UIC-B). Among women with a history suspected for CI, rates of sPTB < 34 weeks were 13% lower after HIC than after UIC-A, with lower rates of PPROM, (suspected) infection, and admission for threatened sPTB. In women receiving UIC, a history suspected for CI (UIC-A) was associated with higher rates of sPTB < 34 weeks and PPROM, whereas most other outcomes were comparable between groups. Post-cerclage cervical length and distance from the cerclage to the external os were significantly associated with GA at delivery and pregnancy prolongation.

### 4.2 Interpretation

Preventive strategies for sPTB aim to balance early intervention with avoiding unnecessary procedures. In our cohort, women with a history suspected for CI who received a HIC had a comparable GA at delivery compared to UIC-A (38.4 versus 37.9 weeks, respectively). However, the sPTB rate < 34 weeks of gestation was 13% lower after HIC than UIC-A, while peri- and post-operative complications were very low. These differences should be interpreted in the context of confounding by indication, as women requiring UIC had already developed cervical shortening and therefore represent a clinically distinct, higher-risk group. Our findings are in line with previous research showing reduced sPTB, low birthweight, NICU-admission, and chorioamnionitis after HIC compared with UIC, with similar miscarriage and perinatal mortality rates [25]. However, variation in obstetric history definitions across studies limits direct comparison. Our findings differ from systematic reviews comparing HIC with conservative management (ultrasonography surveillance with UIC placement only upon cervical shortening), which reported comparable sPTB rates [[15], [16], [17],^26^]. These differences likely reflect variation in study populations: women were included with previous sPTB between 30–37 weeks of gestation, whereas our study focused on prior sPTB between 16–28 weeks, more suggestive of CI. Consequently, direct comparison is not feasible. Taken together, these findings suggest that observed differences between HIC and UIC in women with an obstetric history indicative of CI likely reflect differences in underlying risk profile and clinical presentation, although a contribution of treatment strategy cannot be excluded. This underscores the importance of careful risk stratification when selecting and counselling women for cerclage.

Obstetric history is a critical determinant of pregnancy outcome. After UIC, median GA at delivery was 38.0 weeks with a prolongation of pregnancy after cerclage placement 116.0 days, consistent with previous reports [18,[27], [28], [29], [30], [31]]. Our sPTB rates < 34 weeks (15%) and < 32 weeks (11%) were lower than previously reported ranges, suggesting relatively favourable outcomes in our population [28,32]. A possible explanation is the uniform surgical technique, with high cerclage placement (20–25 mm from the external os) after bladder mobilisation. Higher placement may improve mechanical support and reduce sPTB risk [32,33]. When stratified by obstetric history, most outcomes remained comparable between women with and without a history suggestive for CI. However, women receiving UIC with a history suspected for CI showed higher rates of sPTB < 34 weeks and PPROM. This group was heterogeneous due to variation in referral patterns and surveillance over time, possibly influencing outcomes. Although few studies have directly compared UIC outcomes between women with and without a history suspected for CI, our findings differ from those of Lavie et al., reporting no significant differences between groups [31]. This discrepancy may stem from differing definitions of obstetric history, as they used a broader criterion of sPTB < 37 weeks compared with our stricter definition of 16–28 weeks. In contrast, our results are consistent with studies reporting sPTB rates < 34–35 weeks of 28–32% in women with a history of sPTB versus 20% in those without. [9,29], Taken together, these findings suggest that in women receiving UIC, presence of an obstetric history suspected for CI confers an elevated risk of sPTB < 34 weeks of gestation and should prompt closer monitoring.

Cervical length is a key predictor of sPTB. In our cohort, post-cerclage distance from the cerclage to external os was significantly associated with GA at delivery and pregnancy prolongation in women receiving either HIC or UIC, and post-cerclage total cervical length was associated with both in women receiving UIC (Fig. 1). In contrast, neither the extent nor rate of cervical shortening pre-UIC was associated with outcomes, and total pre-cerclage cervical length was not associated with GA at delivery. These observations are consistent with previous studies suggesting that pre-cerclage measurements are less informative, whereas post-cerclage values provide more meaningful prognostic information [32,34,35]. Our data highlight the clinical importance of routine post-cerclage cervical assessment, which is not standard practice in many centres. Systematic measurement after cerclage allows early identification of women at higher risk of sPTB, supports timely surveillance or additional interventions, thereby providing clinically relevant prognostic information regarding pregnancy outcomes. Previous research also indicates that in women requiring UIC, a persistently short cervix or further shortening post-cerclage (for example to ≤15 mm) is associated with substantially higher risk of sPTB, low birthweight, and neonatal morbidity, while thresholds are less discriminative in HIC [34,35]. A recent prospective multicentre study similarly evaluated predictors of pregnancy outcome after cerclage, but postoperative cervical measurements and cerclage position could not be assessed as postoperative transvaginal ultrasound was not routinely performed [36]. Our findings highlight the post-cerclage distance from the external os to the cerclage as a clinically relevant and potentially modifiable factor associated with pregnancy outcome.

### 4.3 Strength and limitations

This study has several strengths. Our analyses included a relatively large cohort of women. Stratifying participants according to cerclage indication reduced overall heterogeneity. Well-defined inclusion criteria and standardized surgical/perioperative protocols enhanced internal validity and reproducibility. Cervical measurements pre- and post-cerclage were systematically documented, enabling evaluation of predictive factors. Strict inclusion criteria ensured a clear distinction between cerclage indications. Robust outcome measures, including maternal and neonatal parameters, coupled with regression- and subgroup-analyses, provided a thorough evaluation of key prognostic factors.

However, some limitations need addressing. First, the retrospective design limits causal inference, introduces potential selection bias, and is dependent on availability and completeness of documented information. However, key clinical and cervical measurements were consistently recorded and available, ensuring that analyses were based on robust data. Neonatal morbidity outcomes were not systematically collected and could therefore not be reliably assessed. Secondly, as treatment allocation (HIC versus UIC) was not randomized, comparisons between groups are subject to confounding by indication, meaning observed differences may reflect patient characteristics rather than treatment effects. Moreover, inclusion of women with a previous successful transvaginal cerclage in the HIC group may have selected for women with a more favourable prognosis in subsequent pregnancies. Thirdly, the single-center nature may reduce generalizability to other clinical settings. Furthermore, absence of a control group restricts direct comparisons to alternative management strategies, including close ultrasound surveillance. Not all women underwent serial cervical length surveillance pre-cerclage, reflecting referrals and evolving protocols, which may have introduced heterogeneity. Consequently, direct comparison with expectant management is not possible. Furthermore, data regarding concurrent progesterone use could only be reliably retrieved for a limited proportion of the cohort and were therefore insufficient to assess its distribution across groups or include progesterone use in the analyses; residual confounding by progesterone treatment therefore cannot be excluded. Additionally, although standardized surgical protocols were applied, some residual variation in technique and postoperative care cannot be excluded. Furthermore, formal assessment of the assumptions underlying the exploratory linear regression analyses was not performed, and these results should therefore be interpreted with caution. Finally, multiple subgroup and exploratory analyses were performed, increasing the risk of type I error.

## 5 Conclusion

In this 11-year observational cohort, median GA at delivery was comparable between women receiving HIC and UIC. However, in women with a history suspected for CI (sPTB <28 weeks), women receiving HIC had lower rates of sPTB < 34 weeks and pregnancy complications compared with UIC. In women with an UIC, having a history suspected for CI was associated with higher risk of sPTB in the current pregnancy. For both HIC and UIC, greater distance between the cerclage and external os was associated with higher GA at delivery and greater pregnancy prolongation, identifying cerclage height as a clinically relevant and modifiable factor. Together, these findings suggest that pregnancy outcome after cerclage is determined not only by patient selection and clinical context, but also by procedural factors, underscoring the importance of individualized decision-making combined with optimal cerclage placement.

## CRediT authorship contribution statement

**Cecile C. Hulshoff:** Writing – review & editing, Writing – original draft, Visualization, Methodology, Investigation, Formal analysis, Data curation, Conceptualization. **Maria G. Plasman:** Writing – original draft, Methodology, Investigation, Formal analysis, Data curation. **Kanthi de Groot:** Writing – original draft, Methodology, Investigation, Formal analysis, Data curation. **Jacklijn A.A. Pijls:** Writing – review & editing, Methodology, Investigation, Formal analysis, Data curation. **Freke A. Wilmink:** Writing – review & editing, Methodology. **Marc E.A. Spaanderman:** Writing – review & editing, Methodology, Investigation, Formal analysis, Data curation. **Ralph R. Scholten:** Writing – review & editing, Methodology, Conceptualization. **Joris van Drongelen:** Writing – review & editing, Supervision, Project administration, Methodology, Funding acquisition, Conceptualization.

## Ethics statement

Our institutional medical ethical review board considered this study not to be human subjects research and exempted it from review on April 9, 2023 (reference number 2023–16264).

## Funding

none.

## Declaration of Competing Interest

The authors declare that they have no known competing financial interests or personal relationships that could have appeared to influence the work reported in this paper.
