# Supplementary material for: Pregnancy outcomes and prognostic factors after history- and ultrasound-indicated cerclage in women with cervical insufficiency and/or short cervix: A retrospective cohort study
Source: Eur J Obstet Gynecol Reprod Biol X. 2026 Sep 7;32:100489. doi: 10.1016/j.eurox.2026.100489 (PMC13586745; doi:10.1016/j.eurox.2026.100489)
Supplement: Supplementary file 1 [file mmc1.docx]

***Appendices***

*Appendix A: Kaplan Meier curve of the percentage of women remaining pregnant during the course of pregnancy after placement of the ultrasound-indicated cerclage stratified by pre-cerclage cervical length*

*Appendix B: Pregnancy outcomes after placement of the ultrasound-indicated cerclage for cervical length ≤15 mm and >15 mm*

|  | A. With history suspected for CI | | B. Without history suspected for CI | | Total | |
| --- | --- | --- | --- | --- | --- | --- |
|  | ≤15 mm | >15 mm | ≤15 mm | >15 mm | ≤15 mm | >15 mm |
| Number of participants | 14 | 22 | 22 | 21 | 36 | 43 |
| *Obstetric outcomes* |  |  |  |  |  |  |
| GA at delivery (weeks) | 37.1 (4.8) | 37.5 (3.4) | 38.2 (2.0) | 37.3 (3.3) | 37.9 (2.6) | 37.3 (3.4) |
| Delivery ≥ 34 weeks of GA (%) | 10 (71%) | 17 (77%) | 21 (95%) | 19 (90%) | 31 (86%) | 36 (84%) |
| Delivery < 34 weeks of GA (%) | 4 (29%) | 5 (23%) | 1 (5%) | 2 (10%) | 5 (14%) | 7 (16%) |
| Delivery < 32 weeks of GA (%) | 2 (14%) | 4 (18%) | 1 (5%) | 2 (10%) | 3 (8%) | 6 (14%) |
| Delivery < 28 weeks of GA (%) | 0 (0%) | 2 (9%) | 1 (5%) | 1 (5%) | 1 (3%) | 3 (7%) |
| Delivery < 24 weeks of GA (%) | 0 (0%) | 1 (5%) | 0 (0%) | 1 (5%) | 0 (0%) | 2 (5%) |
| Pregnancy prolongation (days) | 114.5 (37.3) | 115.0 (38.5) | 117.0 (16.5) | 112.0 (39.0) | 116.0 (19.5) | 113.0 (40.5) |
| *Neonatal outcomes* |  |  |  |  |  |  |
| Survival rate (%) |  |  |  |  |  |  |
| - Overall: | 14 / 14 (100%) | 21 / 22 (95%) | 22 / 22 (100%) | 20 / 21 (95%) | 36 / 36 (100%) | 41 / 43 (95%) |
| - Fetal survival rate (%) ^a^ | 14 / 14 (100%) | 22 / 22 (100%) | 22 / 22 (100%) | 21 / 21 (100%) | 36 / 36 (100%) | 43 / 43 (100%) |
| - Neonatal survival (%) ^b^ | 14 / 14 (100%) | 21 / 22 (95%) | 22 / 22 (100%) | 20 / 21 (95%) | 36 / 36 (100%) | 41 / 43 (95%) |
| - Fetuses born <24 weeks of GA: | NA | 0 / 1 (0%) | NA | 0 / 1 (0%) | NA | 0 / 2 (0%) |
| - Non-viable fetal survival <24 weeks (%) ^a^ |  | 1 / 1 (100%) |  | 1 / 1 (100%) |  | 2 / 2 (100%) |
| - Non-viable neonatal survival <24 weeks (%) ^b^ |  | 0 / 1 (0%) |  | 0 / 1 (0%) |  | 0 / 2 (0%) |
| - Fetuses born ≥24 weeks of GA: | 14 / 14 (100%) | 21 / 21 (100%) | 22 / 22 (100%) | 20 / 20 (100%) | 36 / 36 (100%) | 41 / 41 (100%) |
| - Viable fetal survival rate ≥24 weeks (%) ^a^ | 14 / 14 (100%) | 21 / 21 (100%) | 22 / 22 (100%) | 20 / 20 (100%) | 36 / 36 (100%) | 41 / 41 (100%) |
| - Viable neonatal survival ≥24 weeks (%) ^b^ | 14 / 14 (100%) | 21 / 21 (100%) | 22 / 22 (100%) | 20 / 20 (100%) | 36 / 36 (100%) | 41 / 41 (100%) |
| GA = gestational age; CI = cervical insufficiency.  Data are presented as median (IQR) or n (%).  Bold indicates statistical significance, with significance set at p-value < 0.05 (1 = A. With history of cervical insufficiency ≤15 mm VS >15 mm. 2 = B. Without history of cervical insufficiency ≤15 mm VS >15 mm. 3 = Total ≤15 mm VS >15 mm).  ^a^ = Based on fetuses alive at birth.  ^b^ = Based on neonates alive at the 28^th^ day after birth. | | | | | | |

*Appendix C: Pregnancy outcomes after placement of the history- or ultrasound-indicated cerclage with a history of cervical insufficiency but no previous
cerclage placement*

|  | **History-indicated cerclage (HIC), no previous cerclage** | **Ultrasound-indicated cerclage (UIC), no previous cerclage** | **p-value** |
| --- | --- | --- | --- |
| **Number of participants** | 153 | 34 |  |
| ***Obstetric outcomes*** | | | |
| **GA at delivery (weeks)** | 38.3 (2.9) | 37.4 (5.2) | 0.22 |
| **Delivery ≥ 34 weeks of GA (%)** | 135 (88%) | 25 (74%) |  |
| **sPTB < 34 weeks of GA (%)** | 18 (12%) | 9 (26%) |  |
| **sPTB < 32 weeks of GA (%)** | 14 (9%) | 6 (18%) |  |
| **sPTB < 28 weeks of GA (%)** | 8 (5%) | 2 (6%) |  |
| **sPTB < 24 weeks of GA (%)** | 3 (2%) | 1 (3%) |  |
| **Neonatal outcomes** | | | |
| **Survival** |  |  |  |
| - **Overall:** | 149 / 153 (97%) | 33 / 34 (97%) | 1.00 |
| - **Fetal survival rate (%) ^a^** | 151 / 153 (99%) | 33 / 34 (97%) | 1.00 |
| - **Neonatal survival (%) ^b^** | 149 / 151 (99%) | 33 / 33 (100%) | 1.00 |
| - **Fetuses born <24 weeks of GA:** | 0 / 3 (0%) | 0 / 1 (0%) | 1.00 |
| - **Non-viable fetal survival <24 weeks (%) ^a^** | 1 / 3 (33%) | 0 / 1 (0%) | 1.00 |
| - **Non-viable neonatal survival <24 weeks (%) ^b^** | 0 / 1 (0%) | NA | 1.00 |
| - **Fetuses born ≥24 weeks of GA:** | 149 / 150 (99%) | 33 / 33 (100%) | 1.00 |
| - **Viable fetal survival rate ≥24 weeks (%) ^a^** | 150 / 150 / (100%) | 33 / 33 (100%) | 1.00 |
| - **Viable neonatal survival ≥24 weeks (%) ^b^** | 149 / 150 (99%) | 33 / 33 (100%) | 1.00 |
| GA = gestational age; sPTB = spontaneous preterm birth.  Data are presented as median (IQR) or n (%). Bold indicates statistical significance, with significance set at p-value < 0.05.  ^a^ = Based on fetuses alive at birth.  ^b^ = Based on neonates alive at the 28^th^ day after birth. | | | |

*Appendix D: Figure 1 Kaplan Meier curve of the percentage of women remaining pregnant during the course of pregnancy after placement of the history- or ultrasound-indicated cerclage with a history of cervical insufficiency but no previous cerclage placement*

*Appendix E: Multivariable linear regression of pre-cerclage cervical length change and measurement interval on gestational age at delivery in women with a ultrasound-indicated cerclage*

|  | **UIC total** | | | **UIC with history of cervical insufficiency** | | | **UIC without history of cervical insufficiency** | | |
| --- | --- | --- | --- | --- | --- | --- | --- | --- | --- |
|  | **β (95% CI)** | **SE** | **p-value** | **β (95% CI)** | **SE** | **p-value** | **β (95% CI)** | **SE** | **p-value** |
| **Model 1: Change in cervical length and interval before cerclage** | | | | | | | | | |
| Intercept | 36.53 (35.28 ; 37.78) | 0.63 | <0.01 | 35.36 (33.24 ; 37.47) | 1.04 | <0.01 | 37.58 (36.13 ; 39.04) | 0.72 | <0.01 |
| Change in cervical length (mm) | -0.05 (-0.18 ; 0.08) | 0.06 | 0.43 | -0.09 (-0.31 ; 0.13) | 0.11 | 0.43 | -0.03 (-0.17 ; 0.11) | 0.07 | 0.67 |
| Time interval between measurements (weeks) | -0.03 (-0.35 ; 0.29) | 0.16 | 0.85 | -0.03 (-0.59 ; 0.52) | 0.27 | 0.90 | -0.05 (-0.43 ; 0.32) | 0.18 | 0.77 |
| **Model 2: Interaction between change in cervical length and interval before cerclage** | | | | | | | | | |
| Intercept | 36.44 (35.12 ; 37.76) | 0.66 | <0.01 | 35.55 (33.30 ; 37.79) | 1.10 | <0.01 | 37.25 (35.75 ; 38.74) | 0.73 | <0.01 |
| Change in cervical length (mm) | -0.07 (-0.24 ; 0.09) | 0.08 | 0.36 | -0.05 (-0.31 ; 0.22) | 0.13 | 0.73 | -0.14 (-0.34 ; 0.06) | 0.10 | 0.16 |
| Time interval between measurements (weeks) | 0.06 (-0.45 ; 0.58) | 0.26 | 0.81 | -0.26 (-1.23 ; 0.70) | 0.47 | 0.58 | 0.27 (-0.28 ; 0.81) | 0.27 | 0.33 |
| Interaction: change in cervical length × Time interval | 0.01 (-0.02 ; 0.04) | 0.01 | 0.64 | -0.01 (-0.06 ; 0.03) | 0.02 | 0.56 | 0.03 (-0.01 ; 0.06) | 0.02 | 0.12 |
| **Model 3: Rate of cervical shortening per week before cerclage** | | | | | | | | | |
| Intercept | 36.95 (35.87 ; 38.03) | 0.54 | <0.01 | 35.63 (33.35 ; 37.90) | 1.10 | <0.01 | 37.67 (36.50 ; 38.85) | 0.58 | <0.01 |
| Change in cervical length per week (mm/week) | -0.02 (-0.06 ; 0.03) | 0.02 | 0.48 | -0.19 (-0.63 ; 0.25) | 0.21 | 0.38 | -0.01 (-0.04 ; 0.03) | 0.02 | 0.57 |
| **Model 4: Baseline cervical length, change, and interval before cerclage** | | | | | | | | | |
| Intercept | 37.52 (34.62 ; 40.42) | 1.45 | <0.01 | 35.27 (30.23 ; 40.32) | 2.47 | <0.01 | 39.05 (35.64 ; 42.47) | 1.68 | <0.01 |
| Baseline cervical length at GA 10-12 weeks (mm) | -0.06 (-0.21 ; 0.10) | 0.08 | 0.46 | 0.00 (-0.25 ; 0.26) | 0.12 | 0.97 | -0.09 (-0.29 ; 0.10) | 0.10 | 0.34 |
| Change in cervical length (mm) | -0.10 (-0.27 ; 0.08) | 0.09 | 0.28 | -0.08 (-0.39 ; 0.23) | 0.15 | 0.59 | -0.10 (-0.30 ; 0.11) | 0.10 | 0.33 |
| Time interval between measurements (weeks) | -0.00 (-0.33 ; 0.33) | 0.17 | 0.99 | -0.04 (-0.61 ; 0.53) | 0.28 | 0.90 | 0.01 (-0.39 ; 0.41) | 0.20 | 0.96 |
| UIC = ultrasound-indicated cerclage; GA = gestational age at delivery.  Model 1: Change in cervical length and interval before cerclage: Assesses the association between the absolute change in cervical length prior to cerclage and gestational age at delivery, adjusted for the time interval between measurements.  Model 2: Interaction between change in cervical length and interval before cerclage: Includes an interaction term between the absolute change in cervical length and the time interval between measurements to evaluate whether the timing of cervical shortening modifies the association with gestational age at delivery.  Model 3: Rate of cervical shortening per week before cerclage: Evaluates the association between the rate of cervical shortening per week prior to cerclage and gestational age at delivery.  Model 4: Baseline cervical length, change, and interval before cerclage: Incorporates baseline cervical length (measured at 10–12 weeks of gestation), the absolute change in cervical length, and the time interval between measurements to assess their combined association with gestational age at delivery. | | | | | | | | | |
